# Blood pressure screening in midlife aids in prediction of dementia later in life

**DOI:** 10.48101/ujms.v127.7860

**Published:** 2022-01-03

**Authors:** Linn Moberg, Jerzy Leppert, Simon Liljeström, Mattias Rehn, Lena Kilander, Abbas Chabok

**Affiliations:** a Centre for Clinical Research, Uppsala University, Västmanland County Hospital, Västerås, Sweden; b Department of Public Health and Caring Sciences/Geriatrics, Uppsala University, Uppsala, Sweden

**Keywords:** Midlife hypertension, screening, dementia, prediction, long-term follow-up, prevalence

## Abstract

**Background:**

There is substantial evidence that midlife hypertension is a risk factor for late life dementia. Our aim was to investigate if even high blood pressure at a single timepoint in midlife can predict an increased risk for all-cause dementia, Alzheimer’s disease (AD), or vascular dementia (VaD) later in life.

**Methods:**

The community-based study population comprised 30,102 dementia-free individuals from the *Westmannia Cardiovascular Risk Factors Study*. The participants were aged 40 or 50 years when the health examination took place in 1990–2000. Diagnose registers from both hospitals and primary healthcare centers were used to identify individuals who after inclusion to the study developed dementia. The association between midlife high blood pressure (defined as systolic blood pressure >140 and/or diastolic blood pressure >90 mmHg) at a single timepoint and dementia was adjusted for age, gender, body mass index (BMI), fasting blood glucose, education, smoking, and physical activity level. Multivariate binary cox regression analyses were used.

**Results:**

After a mean follow-up time of 24 years resulting in 662,244 person/years, 761 (2.5%) individuals had been diagnosed with dementia. Midlife high blood pressure at a single timepoint predicted all-cause dementia (hazard ratio [HR]: 1.22, 95% confidence interval [CI]: 1.02–1.45) and VaD (HR: 2.10, 95% CI: 1.47–3.00) but not AD (HR: 1.06, 95% CI: 0.81–1.38).

**Conclusion:**

This study suggests that even midlife high blood pressure at a single timepoint predicts all-cause dementia and more than doubles the risk for VaD later in life independently of established confounders. Even though there was no such association with AD, this strengthens the importance of midlife health examinations in order to identify individuals with hypertension and initiate treatment.

## Introduction

The prevalence of dementia is increasing worldwide, and as of today, there is no disease-modifiable treatment (1–5). This fact has increased the interest in prevention, and potentially modifiable midlife vascular risk factors have been identified (2, 3, 5, 6). There are several subtypes of dementia where Alzheimer’s disease (AD) is the most common followed by vascular dementia (VaD), and altogether, they account for 80% of all dementia cases (5). Coexisting cerebrovascular disease lowers the threshold for AD to become clinically manifest. Thus, both subtypes are associated with vascular risk factors (7).

Midlife hypertension has been highlighted since research indicates that antihypertensive treatment lowers the incidence of dementia later in life (8–10). Also, a number of observational cohort studies have confirmed that midlife hypertension (age 40–64) increases the risk for dementia or cognitive decline later in life (11–17). Most of these studies measured blood pressure repeatedly throughout life and did not differentiate between various dementia subtypes.

Screening for cardiovascular risk factors was highlighted during the 1980–1990s in the western countries. The county of Västmanland, situated near Stockholm, Sweden, started, in 1990, a screening program in a large population: the *Westmannia Cardiovascular Risk Factors Study* (WICTORY) with the overall aim of lowering the cardiovascular mortality rate in the county.

With data from this cohort, we aimed to investigate whether the presence of high blood pressure at a single timepoint at a health examination in midlife is a predictive factor for the development of all-cause dementia, AD, or VaD later in life.

## Materials and methods

### Study population

**Figure 1:**
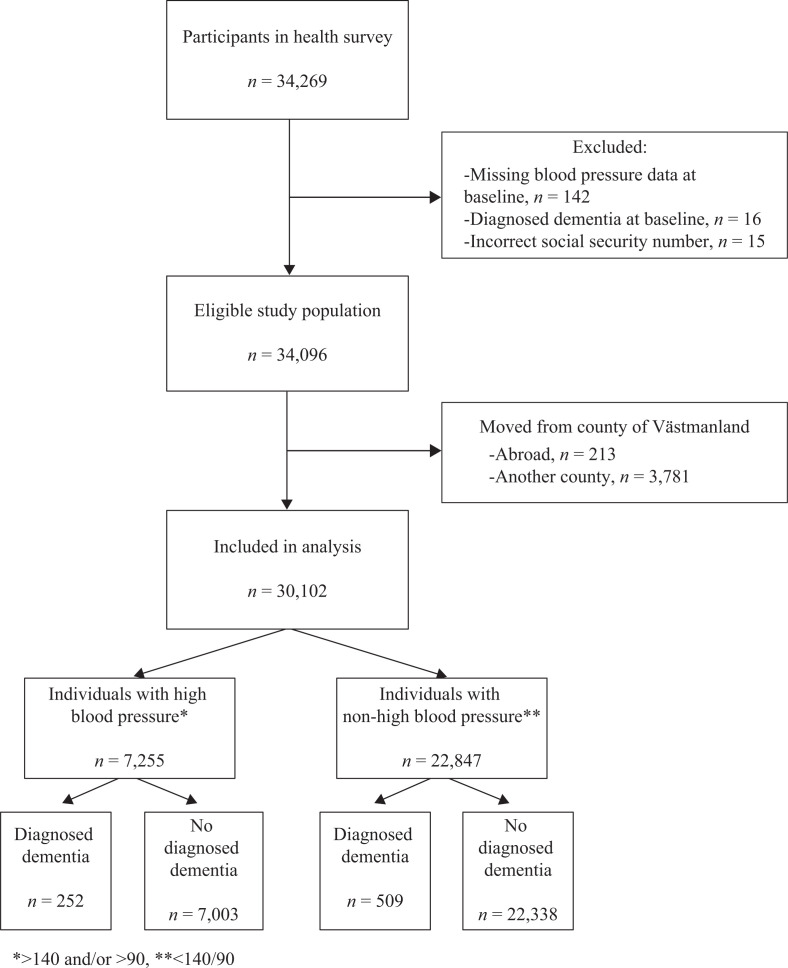
Flow chart of study population.

The intention of WICTORY was to offer a free screening for cardiovascular risk factors to all inhabitants aged 40 or 50 during 1990–2000 (born 1940–1959) and residing in the county of Västmanland. Of the 56,977 eligible individuals, 34,269 (60%) completed the health examination. All participants gave an informed consent. A detailed overview of the selection procedure for the present study is shown in Figure 1. Reasons for exclusion were as follows: missing records of systolic or diastolic blood pressure (DBP), previouly recorded dementia diagnosis, or incorrect social security numbers (PINs). Finally, individuals who had emigrated or moved to another Swedish county (source: Swedish Tax Agency Population Registration) were excluded if they did not already have a record of dementia in our registers, since we were unable to know if they got a dementia diagnosis elsewhere than in Västmanland in 2008 or later (our national registers reach 2007, see ‘diagnoses of dementia’ below). In total, 4,167 individuals were excluded, presented as lost to follow-up in the following text. The final study population comprised 30,102 dementia-free individuals from the WICTORY cohort with age range 60–80 years and mean follow-up time of 24 years. The Uppsala Regional Ethical Review Board approved the study by waiving informed consent (Dnr. 2007/165).

### Baseline examinations

The screening procedure has been presented in detail elsewhere (18). Briefly, data for the following measurements from the WICTORY cohort health examination in 1990–2000 were collected for this study: office blood pressure, body mass index (BMI), fasting blood glucose (fB-glucose), educational level, and smoking and physical activity habits. Blood pressure was measured after 15 min of rest in a sitting position in the upper arm. A manual sphygmomanometer at a suitable size was used, and the average of two measurements was documented. BMI was calculated as the body mass divided by the square of the body height (kg/m^2^). Blood glucose was measured after 2 h refraining from eating and was analyzed using a factory-calibrated photometer. Education was self-reported as number of years post-elementary school. Smoking habits were self-reported in a questionnaire with five alternatives (never, previous, 1–14 cig/day, 15–25 cig/day, and >25 cig/day). Physical activity level was self-reported and defined as low impact exercise for at least 30 min, and a five-grade scale was used (exercising daily, 3–4 times/week, 1–2 times/week, 1–2 times/month, and exercising rarely or never).

### Diagnoses of dementia

The DUVA (Datalager för uppföljning och verksamhetsanalys), 2008–2020, was our main diagnoses register since it comprises data from both primary healthcare centers and hospitals in Västmanland county. In addition, hospital diagnoses registers from the National Board of Health and Welfare (NBHW) were used for the years 1963–2007. Individuals from the cohort were considered to have a diagnosis of dementia if they had a record of any of the ICD-10-SE diagnoses of dementia shown in Table 1. Since ICD-10-SE was implemented in 1997, a conversion of the diagnoses to ICD-9-SE was made to be used for the earlier registers. For this, conversion tables supplied by the NBHW were applied, and included ICD-9-SE diagnoses are also shown in Table 1. Finally, the national register for death causes was used. Both primary and secondary diagnoses were searched for in all registers. Every visit to a doctor where a dementia diagnosis was recorded had been documented. Thus, many individuals had repetitions of diagnoses and sometimes subtypes of dementia. Individuals were included in analyses for AD or VaD if they ever had a record of any of the diagnoses mentioned.

**Table 1:** Dementia diagnoses.

**Vascular dementia**	F01, F010, F011, F012, F013, F018, F019
**Alzheimer’s disease**	F000, F001, F002, F009, G30, G300, G301, G308, G309
**Other dementia types**	F020, F021, F022, F023, F024, F028, F03-P, F039, F051, F107A, G310, G318A
**ICD-9-SE**	290.0, 290.1, 290.2, 290.3, 290.4, 290.8, 290.9, 291.2, 331.0, 331.1

### Statistical analysis

High blood pressure was defined as systolic blood pressure (SBP) >140 mmHg and/or DBP >90 mmHg. Individuals were dichotomized into a high blood pressure group and a non-high blood pressure group (reference category). Regarding confounders, continuous variables were used for age, BMI, and fB-glucose. Others were dichotomized into previous/current smoker or non-smoker (reference), physical activity less than one time per week/never or at least one time per week (reference), and educational level ≤1 year or >1 year of education after elementary school (reference). Baseline characteristics were analyzed using independent sample *t*-tests (continuous variables) and chi-square tests (categorical variables).

The association between midlife high blood pressure and dementia was first analyzed using univariate Cox regression models, with all-cause dementia, VaD, and AD as dependent variables and blood pressure as independent variable. After univariate analysis, multivariate Cox regression models were used to further investigate the effect of putative confounding variables. Dementia was used as dependent variable with blood pressure, BMI, fB-glucose, smoking, physical activity, educational level, age at inclusion, and gender as independent variables. A series of sensitivity analyses were conducted to test the robustness of the results. Altered cut-offs were used (SBP > 130 mmHg and/or DBP > 80 mmHg, and SBP > 150 mmHg and/or DBP 100 mmHg) and analyzed with multivariate Cox regression models. All statistical analyses were performed using IBM SPSS version 26, with *p*-values ≤ 0.05 considered statistically significant.

## Results

Out of the 30,102 individuals included in the analysis, 7,255 (24.1%) had values defined as high blood pressure at the index investigation. After a mean follow-up time of 24 years (which resulted in 662,244 person/years), a total of 761 (2.5%) individuals were diagnosed with dementia: 252 in the high blood pressure group and 509 in the non-high blood pressure group. Mean age for first diagnosis of dementia was 71 years.

Participants who later were diagnosed with dementia had a higher mean age at inclusion and overall, a more accentuated risk profile including significantly higher SBP, DBP, BMI, fB-glucose, and lower education (Table 2). They also had a higher mortality compared to the group with no dementia, 32% versus 13%, respectively. Data regarding mortality among those lost to follow-up were missing. When comparing by blood pressure status, 18% had died in the high blood pressure group versus 13% in the non-high blood pressure group. Mortality in the total cohort was 14%. The group lost to follow-up had a significantly lower mean age, SBP, DBP, and BMI.

**Table 2:** Baseline characteristics at health examination by later dementia status, overall, and lost to follow up.

Characteristics	Dementia *n* = 761	No dementia *n* = 29,341	*p*-Values Dementia/no dementia	Total *n* = 30,102	Lost to follow-up *n* = 4,167	*p*-Values total/lost to follow-up
Age, mean (SD), years	48.9 (3.3)	45.6 (5.0)	*p* < 0.001	45.7 (5.0)	44.9 (5.0)	*p* < 0.001
Female, *n* (%)	401 (52.7)	15,249 (52.0)	n.s.	15,650 (52.0)	2,133 (51.2)	n.s.
SBP, mean (SD), mmHg	134 (18)	130 (17)	*p* < 0.001	130 (17)	128 (16)	*p* < 0.001
DBP, mean (SD), mmHg	85 (11)	83 (10)	*p* < 0.001	83 (10)	81 (10)	*p* < 0.001
BMI, mean (SD), kg/m^2^	25.9 (3.9)	25.6 (3.9)	*p* < 0.05	25.6 (3.9)	25.4 (3.7)	*p* < 0.01
fB-glucose, mean (SD), mmol/L	5.8 (1.3)	5.6 (1.3)	*p* < 0.001	5.6 (1.3)	5.6 (1.1)	n.s.
Smokers, *n* (%)	248 (32.8)	8,576 (29.7)	n.s.	8,824 (29.7)	1,215 (29.6)	n.s.
Physically active, *n* (%)	623 (84.0)	24,343 (84.8)	n.s.	24,966 (84.8)	3,331 (84.0)	n.s.
Elementary school only (%)	178 (27.6)	4,984 (19.1)	*p* < 0.001	5,162 (19.3)	711 (19.1)	n.s.
Dead, *n* (%)	241 (31.7)	3,924 (13.4)	*p* < 0.001	4,165 (13.8)	–	n.a.

SBP: systolic blood pressure; DBP: diastolic blood pressure; BMI: body mass index; fB-glucose: blood glucose after 2 h fasting; smokers: previous/current smoker; physically active: 30 minutes at least one time/week; n.s.: not significant; n.a.: not available; SD: standard deviation.

Out of the 761 individuals diagnosed with dementia, 166 (22%) had received a diagnosis of VaD and 343 (45%) a diagnosis of AD. Since some individuals had more than one dementia diagnosis, there was an overlap between the two groups. Thirty-seven individuals had a diagnosis of both VaD and AD, resulting in 472 (62%) individuals ever diagnosed with AD or VaD. Unadjusted values of midlife high blood pressure at a single timepoint were associated with a significantly increased risk for all-cause dementia (HR: 1.69, 95% confidence interval [CI]: 1.45–1.96, *p*-value < 0.001), VaD (HR: 2.91, 95% CI: 2.15–3.96, *p*-value < 0.001), and AD (HR: 1.51, 95% CI: 1.20–1.89, *p*-value < 0.001). In the multivariate analysis, midlife high blood pressure at a single timepoint did significantly increase the risk for both all-cause dementia (HR: 1.22, 95% CI: 1.02–1.45, *p*-value 0.026) and VaD (HR: 2.10, 95% CI: 1.47–3.00, *p*-value < 0.001). For AD, there was no statistically significant association (HR: 1.06, 95% CI: 0.81–1.38, *p*-value 0.940). All results are shown in Table 3.

**Table 3:** Cox proportional hazard models of midlife high blood pressure^1^ at a single timepoint and risk for all-cause dementia, vascular dementia, and Alzheimer’s disease later in life.

Dementia type	Unadjusted HR (95% CI)	Adjusted for age at inclusion, gender, education, BMI, fB-glucose, smoking, and physical activity level, HR (95% CI)
All-cause dementia (*n* = 761)	1.69 (1.45–1.96)***	1.22 (1.02–1.45)*
VaD (*n* = 166)	2.91 (2.15–3.96)***	2.10 (1.47–3.00)***
AD (*n* = 343)	1.51 (1.20–1.89)***	1.06 (0.81–1.38)†

CI: confidence interval; AD: Alzheimer’s disease; VaD: vascular dementia; BMI: body mass index; fB-glucose: fasting blood glucose; HR, hazard ratio.

1 SBP >140 mmHg and/or DBP > 90 mmHg.

* *p* < 0.05;

*** *p* < 0.001;

† *n.s*.

Sensitivity analyses with altered cut-offs revealed no major differences from the primary results. Concerning all-cause dementia, adjusted hazard ratios for a lower cut-off (SBP > 130 mmHg and/or DBP > 80 mmHg) was 1.22 (95% CI: 1.03–1.45) and a higher cut-off (SBP > 150 mmHg and/or DBP > 100 mmHg) was 1.34 (95% CI: 1.08–1.67). Sensitivity analyses for VaD revealed adjusted hazard ratios for a lower cut-off was 1.73 (95% CI: 1.16–2.57) and for a higher cut-off = 1.70 (95% CI: 1.11–2.60). Sensitivity analyses for AD were statistically non-significant.

## Discussion

In this community-based prospective cohort study of more than 30,000 individuals with a mean follow-up of 24 years, midlife high blood pressure at a single timepoint predicted all-cause dementia and more than doubled the risk for VaD later in life when adjusted for age, gender, education, and other established midlife vascular risk factors. However, midlife high blood pressure at a single timepoint was not associated with the risk of AD when adjusted for the same confounders.

Hypertension has been suggested to be the largest potentially preventable risk factor for cerebrovascular damage (5). Hypertension changes the structure of the cerebral vessels and consequently in the neurovascular unit in several ways. First, it promotes atherosclerosis in both extra- and intracranial arteries, which, in turn, increases the risk for thromboembolic stroke, intracranial hypoperfusion, and vessel rupture causing hemorrhagic stroke (5, 19). After a stroke, there is a 30% risk to develop dementia (20, 21). Also, silent (asymptomatic) infarcts increase the risk for dementia (20). Midlife high blood pressure has been associated with increased white matter hyperintensity volumes (12), and autoregulation of cerebral blood flow is also disturbed in cases of long-lasting hypertension (5). In addition, hypertension is the main risk factor for microbleeds, which also contribute to the pathogeneses leading to cognitive impairment (5, 22). Together, all these mechanisms contribute to cerebral ischemia, which increases the risk for primarily VaD but also all-cause dementia.

There is emerging evidence of a neuropathological overlap between cerebrovascular disease and AD (7, 19, 23). A neuropathological study has shown that in 40% of clinically diagnosed AD cases, cerebrovascular lesions are present suggested to have lowered the threshold to dementia (7). Considering this, it is surprising that we could not show an association between single value of midlife high blood pressure and AD later in life. Previous cohort studies measuring repeated blood pressures have, however, shown this association (24). On the other hand, some previous cohort studies with repeated blood pressures presented results in line with ours regarding AD (15, 25).

The validity of using registers of diagnoses might be debatable. In this particular study design, we have not examined the patients ourselves as many previous cohort studies have done (13, 15, 16), which may question the validity in the recorded diagnoses. For reference, it has been suggested that generally, 85–95% of all inpatient registered diagnoses are correct (26). However, regarding dementia, this number is suggested to be only 26% (27). In the present study, diagnoses were collected both from hospitals and primary healthcare centers, meaning that the correct number should reasonably be higher since dementia diagnoses most often are identified by primary care physicians. The fact that hospitals in general were included might have resulted in lower numbers of specific dementia diagnoses compared to unspecified ones. Thus, the relatively low proportion of AD and VaD (62% of all-cause dementia) in our study might be false due to unrecorded or misclassified cases. This might have contributed to an underestimation of the effect of midlife high blood pressure at a single timepoint on the development of AD and VaD later in life, but not all-cause dementia. However, this fact alone does probably not explain the unsignificant results regarding AD since its association to hypertension is not as strong as for VaD.

The dementia prevalence of 2.5% in this cohort with individuals aged 60–80 years, mean age 71 for first diagnosis, is considered reliable. According to a Swedish report from 2006, the prevalence of demented individuals aged 65 is 1%, and the figure is expected to be doubled for every 5 years (28).

Moreover, mortality rates in the high blood pressure group were significantly higher than in the total cohort (18% vs. 14%). Considering hypertension being the largest risk factor for cardiovascular disease and all-cause mortality (24), death caused by hypertension might be a competing risk for the possibility of diagnosing dementia.

Strengths of this study include the large community-based population and a long follow-up time. Another strength is our differentiating into dementia subtypes, thus contributing to clarify the associated risk for each subtype. At this period of time, brain computed tomography (CT) scan was recommended in the guidelines for dementia investigation, indicating that the differentiation between vascular and neurodegenerative disorders should be reasonably correct. Furthermore, we were able to adjust for several established midlife confounders in the multivariate analyses, and primary results were confirmed by sensitivity analyses. Continuous variables were used when possible to minimize residual confounding.

There are several limitations to this study. First, we did not take into account whether individuals were on antihypertensive medication at baseline or during follow-up. This information is available from the NBHW in the prescribed drug register starting 2006 but was not retrieved for the present study. It would be beneficial for future cohort studies to separate individuals with high blood pressure at baseline into those who are on antihypertensive treatment and those who are not. Also, it would be of great value to investigate compliance to prescribed medication by using data from the prescribed drugs register. Another source of limitation was that it was clearly obvious in the registers that round offs had been made when measuring blood pressure. This is the reason why we chose >140/90 mmHg and not ≥140/90 mmHg as our definition of high blood pressure. This can also be considered a strength since we, with this definition, probably have minimized the risk of false high blood pressure values. We did not use the term hypertension since this diagnosis cannot be confirmed by measurements from a single timepoint (24). Regarding analysis for VaD and AD, ICD-9 codes were not taken into account since before 1997, differentiated dementia diagnoses were not available. Finally, our results cannot be generalized to other populations because an overwhelming majority of study participants were Scandinavians.

In conclusion, our study suggests that midlife high blood pressure at a single timepoint predicts all-cause dementia and more than doubles the risk for VaD later in life when adjusted for established confounders. Regarding AD, only unadjusted values showed association between midlife high blood pressure at a single timepoint and AD later in life. We were unable to investigate the validity in using diagnosis registers from primary healthcare centers regarding dementia. This would be desirable in future studies. Also, our study design using midlife high blood pressure values from a single time-point has to be replicated in future studies. Learning from these, we could investigate when midlife health examinations should be initiated in order to start treatment at an optimal age.
